# A novel heterozygous missense *MYH7* mutation potentially causes an autosomal dominant form of myosin storage myopathy with dilated cardiomyopathy

**DOI:** 10.1186/s12872-023-03538-8

**Published:** 2023-10-04

**Authors:** Niloofar Naderi, Neda Mohsen-Pour, Yalda Nilipour, Maryam Pourirahim, Majid Maleki, Samira Kalayinia

**Affiliations:** 1 Cardiogenetic Research Center, Rajaie Cardiovascular Medical and Research Center, University of Medical Sciences, Tehran, Iran; 2 Zanjan Pharmaceutical Biotechnology Research Center, Zanjan University of Medical Sciences, Zanjan, Iran; 3 Pediatric pathology research center, Research institute for children’s health, Shahid Beheshti University of Medical Sciences, Tehran, Iran

**Keywords:** *MYH7*, Whole-exome sequencing, Myosin storage myopathy, Dilated cardiomyopathy, Myosin, *In silico*

## Abstract

**Background:**

The *MYH7* gene, which encodes the slow/ß-cardiac myosin heavy chain, is mutated in myosin storage myopathy (MSM). The clinical spectrum of MSM is quite heterogeneous in that it ranges from cardiomyopathies to skeletal myopathies or a combination of both, depending on the affected region. In this study, we performed clinical and molecular examinations of the proband of an Iranian family with MSM in an autosomal dominant condition exhibiting proximal muscle weakness and dilated cardiomyopathy.

**Methods:**

Following thorough clinical and paraclinical examinations, whole-exome sequencing `was performed on the proband (II-5). Pathogenicity prediction of the candidate variant was performed through *in-silico* analysis. Co-segregation analysis of the WES data among the family members was carried out by PCR-based Sanger sequencing.

**Results:**

A novel heterozygous missense variant, *MYH7* (NM_000257): c.C1888A: p.Pro630Thr, was found in the DNA of the proband and his children and confirmed by Sanger sequencing. The *in-silico* analysis revealed that p.Pro630Thr substitution was deleterious. The novel sequence variant fell within a highly conserved region of the head domain. Our findings expand the spectrum of *MYH7* mutations.

**Conclusions:**

This finding could improve genetic counseling and prenatal diagnosis in families with clinical manifestations associated with *MYH7*-related myopathy.

**Supplementary Information:**

The online version contains supplementary material available at 10.1186/s12872-023-03538-8.

## Introduction

Myosin, which is a highly conserved protein in all eukaryotic cells, is not only the major constituent of skeletal muscle thick filaments but also a crucial element for body movement and heart contractility. It contains two elongated globular heads connected to a long helical coiled coil (the myosin rod). This hexameric protein consists of two myosin heavy chain (MyHC) subunits and four light chain subunits. Each head, or subfragment 1 (S1), is comprised of approximately 850 N-terminal residues of one MyHC and one of each light chain. The heads, including actin and ATP-binding regions, are liable for the force transduction properties of myosin [1]. The N-terminal region of the myosin rod, designated as subfragment 2 (S2), joins S1 to the filament backbone. The myosin rod is a parallel α-helical coiled-coil dimer of the C-terminal of MyHC tails. The larger C-terminal part of the rod, named “light meromyosin (LMM)”, lies along the thick filament axis and mediates filament assembly [2]. The LMM also provides sites for the binding of myosin-associated proteins like myomesin 1, myosin-binding protein C and H, M-protein, and titin.

There are three major MyHC isoforms expressed in human limb skeletal muscles. MyHC IIx, coded by *MYH1* as a member of the *MYH* gene family, is expressed in fast, glycolytic, type 2B muscle fibers. MyHC IIa, encoded by *MYH2*, is expressed in fast, intermediate, type 2 A muscle fibers. Slow/ß-cardiac MyHC (MyHC I), encoded by the *MYH7* gene, is expressed in slow, oxidative, type 1 muscle fibers. It is also expressed in the ventricles of the heart [3]. Located on chromosome 14, the *MYH7* gene (OMIM # 160,760) contains approximately 22,883 bp, including 41 exons [4]. Pathogenic mutations in *MYH7* have been reported to cause a wide range of clinical expressions ranging from hereditary skeletal muscle diseases, including Laing distal myopathy [5] and myosin storage myopathy (MSM) with or without cardiac involvement, to isolated cardiomyopathies such as dilated cardiomyopathy [6], hypertrophic cardiomyopathy [7], and left ventricular non-compaction cardiomyopathy [8], depending on the residue of *MYH7* that is affected [9]. *MYH7* mutations are reported in 14–25% of all cardiomyopathy cases [10].

MSM (OMIM #608,358), also known as “hyaline body myopathy”, is a rare, congenital myopathy identified by subsarcolemmal accumulations of myosin in type 1 skeletal muscle fibers resulting in the weakness of the scapula, limb, and distal muscles. This myopathy was first described by Cancilla et al. [11] as “familial myopathy with probable lysis of myofibrils of type I fibers” in 1971. Following the molecular nosologic identification of the mutation Arg1845Trp in the rod region of *MYH7*, Tajsharghi et al. [1] proposed the unifying term “myosin storage myopathy” for this disease in 2003. Although the disease is inherited in an autosomal dominant or recessive fashion [12–14], a few sporadic cases with no previous family history have been reported [1, 15]. The onset is generally in childhood, but it may be manifested much later in middle age. Mutations causing MSM are located in the distal end of the tail of MyHC I (exons 37–40 of *MYH7*) [3]. The clinical manifestations of the disease are highly variable among affected individuals, ranging from no weakness to severe impairment of ambulation [1, 9, 11, 15–23] even within the same family [9, 17, 20]. Further, it has been reported that many patients with MSM suffer from delayed motor milestones and usually present with proximal muscle weakness in the four limbs, difficulties in climbing stairs, or running and waddling gait [24].

Given that the conventional approaches to the study of gene mutations are time-consuming and costly, currently, the next-generation sequencing [25]-based method has been widely used to identify the causative variants of many single-gene disorders. We herein describe an Iranian family with an autosomal dominantly inherited pattern of MSM presenting with slowly progressive muscle weakness and dilated cardiomyopathy associated with the *MYH7* (NM_000257): c.C1888A: p.Pro630Thr disclosed by whole-exome sequencing. This family remarkably widens the genotypic and phenotypic variability of MSM, manifesting the first report of this variant in *MYH7*-related myopathy with a somewhat distinct phenotype from Iran. The identification of disease-causing variants in a particular population plays an important role in the development of the molecular diagnosis of such disorders.

## Materials and methods

### Ethics approval and consent to participate

The present study was performed in accordance with the Declaration of Helsinki and approved by Rajaie Cardiovascular Medical and Research Center (approval number: IR.RHC.REC.1399.019). Written informed consent was obtained from all participants for their participation and publication of this report.

### Family recruitment and clinical presentations

**Fig. 1:**
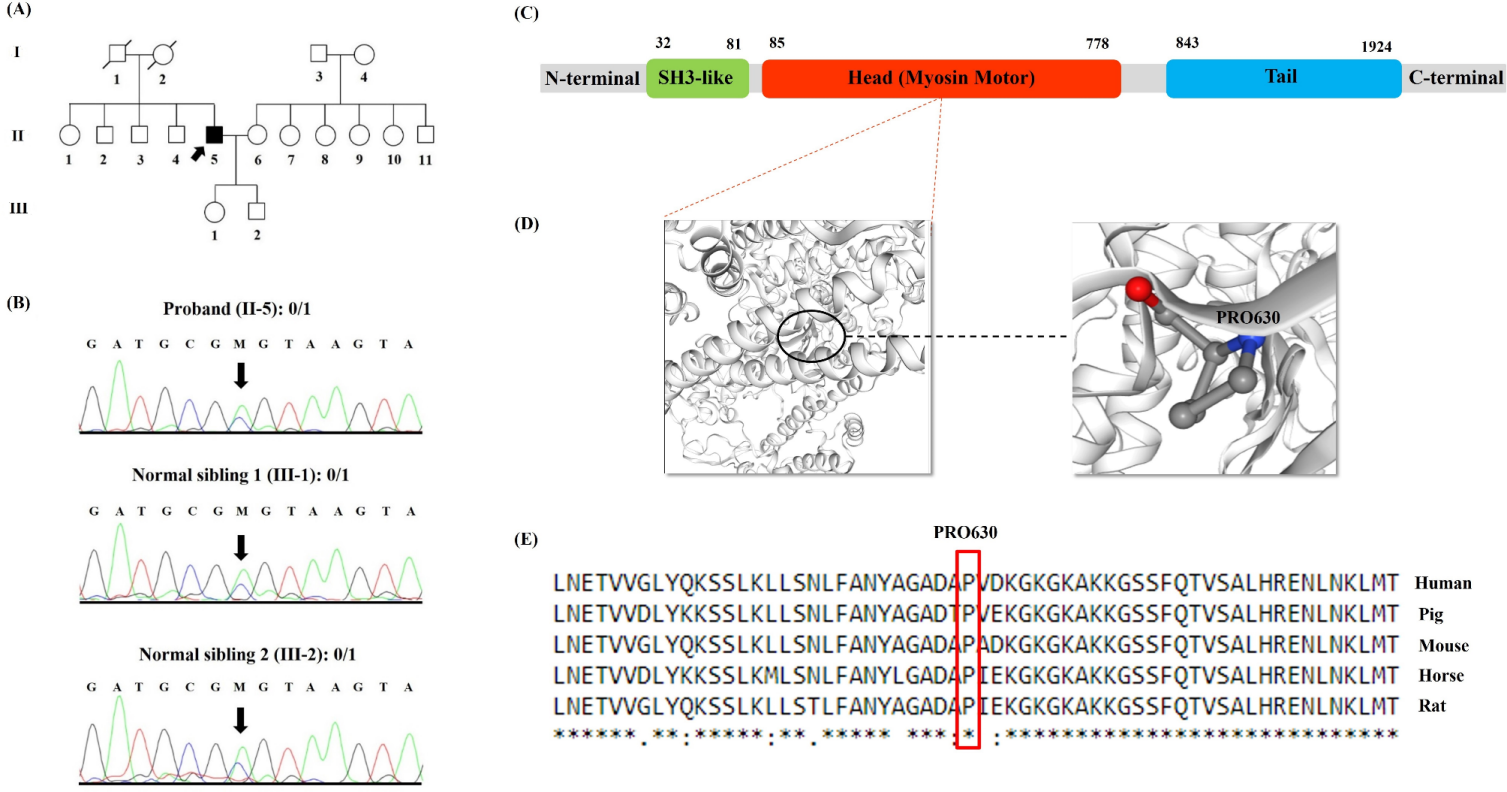
The image presents pedigree, sequencing chromatograms, myosin structure, and conservation analysis in a family affected by *MYH7* mutation. **(A)** Family pedigree of an Iranian family with myopathy: The family investigated in this study consists of 3 generations and 17 members. Only the proband (II-5) is affected (pointed with an arrow). **(B)** The snapshot of the sequencing reads: The proband (II-5), his daughter (III-1), and his son (III-2) carry the c.C1888A: p.Pro630Thr variant in a heterozygous status. The black arrow shows the location of the mutated nucleotide. **(C)** The illustration of the myosin domains. **(D)** The 3D structure of the native and mutated myosin was built using UniProt (https://www.uniprot.org/). The location of the p.Pro630Thr variant is shown on the head portion of myosin. **(E)** The evaluation of the amino-acid evolutionary conservation using CLUSTALW (https://www.genome.jp/tools-bin/clustalw): As depicted, the position of this mutation is highly conserved during evolution

Three generations of an Iranian family recruited for this study are presented in Fig. 1A. The proband (II-5) was a 51-year-old man, who was described as being healthy until age 47. He stated that he had experienced signs of slowly progressive muscle weakness, heart rhythm problems, and extreme shortness of breath since age 47 years. These were worsened during the following four years. Despite the presence of muscle weakness, fatigue, and exercise intolerance with respiratory distress, he had no accurate neuromuscular or cardiological investigation up to the age of 50. A history of taking a statin, which was discontinued, was reported by him. The proband (II-5) had an asymptomatic daughter (Fig. 1A: III-1, 21 years of age), an asymptomatic son (Fig. 1A: III-2, 15 years of age), and also four siblings (II-1, II-2, II-3, and II-4 aged 64, 61, 58, and 55 years, respectively) all without symptoms of muscle or cardiac disease. Unfortunately, the individuals II-1, II-2, II-3, and II-4 were not available to study the clinical and genetic status. Physical examination showed the presence of proximal weakness in the lower limbs and mild scapular winging. He also had some difficulty climbing stairs and lifting his arms above his head. In terms of diagnostic studies, at 51 years of age, the creatine phosphokinase level was elevated to 634 U/L (reference: 0–195). Additionally, high levels of lactate dehydrogenase (295 U/L; normal: 135–225 U/L) and aldolase (7.5 IU/L; normal: 1.5–8.1 IU/L) were found in the proband (II-5). The proband (II-5) was positive for Mi-2a (1+), PL-7 (2+), and RO-52 (2+) in a specific myositis panel. He underwent an extensive clinical investigation, including Pompe disease (MIM #232,300) screening, spiral multi-slice lung computed tomography (CT) scanning, electromyography/nerve conduction studies, muscle biopsies, and magnetic resonance imaging (MRI) on both thighs. Muscle biopsy was performed by open technique and the sample was frozen in isopentane cooled in liquid nitrogen. Frozen sections were stained by Hematoxylin and eosin, Modified Gomori Trichrome, PAS, PAS + diastasis, Oil red O, Congo red, NADH-TR, SDH, COX, COX + SDH and ATPase reactions. These workups led to a diagnosis of myopathy in the proband (II-5).

### Whole-exome sequencing

To determine an accurate mutational diagnosis of myopathy in this family, WES was implemented just on the proband (II-5). Exome was captured using the Agilent SureSelect Exome Capture kit (Agilent Inc, Santa Clara, California, USA). Then, the sequencing of the enriched exon libraries was performed on the Illumina HiSeq 4000 (Macrogen Inc, Seoul, South Korea). The sequencing reads were aligned to the human genome reference (GRCh37 build) by the BWA (v07.17) tool [26]. Next, single-nucleotide polymorphisms/small insertion and deletion (SNP/InDel) was called by applying the GATK (v4.1.4.1) tool with the result file of mapping (BAM). Marking and removing duplicates were performed by SAMtools (in GATK package) [27], followed by recalibration and SNP/InDel calling. For filtering and prioritization, the variants with a minor allele frequency (MAF) more than 0.05 in the 1000 Genomes Project, gnomAD (v2.1.1), and ExAC databases [25] were removed. Prediction tools such as CADD, SIFT, PolyPhen-2, PROVEAN, FATHMM, and GERP^++^ were used for predictive analytics. Accordingly, the variant that was interpreted to be pathogenic in at least four algorithms was considered for confirmation/segregation analysis.

### Polymerase chain reaction (PCR) and segregation analysis

The variant of the *MYH7* gene was sequenced by the PCR and Sanger sequencing method. The primer pairs were designed and validated using Primer3 (v.04.0) (http://bioinfo.ut.ee/primer3-0.4.0/) and BLAST (https://www.ncbi.nlm.nih.gov/tools/primer-blast/index.cgi?LINK_LOC=BlastHome). forward: 5’TATATTGACCATAGAGCAGAA3’ and reverse: 5’TTGCCCTTCTCAATAGCTGCAG3’. PCR was performed on a SimpliAmp™ Thermal Cycler (Thermo Fisher Scientific), with 100 ng DNA, 10pmol/L of primers, 1.5 mmol/L of MgCl2, 200 mmol/L of dNTP, and 1 U Taq DNA polymerase (Amplicon, UK). Then incubation was carried out at 95 °C for 5 min, 35 amplification cycle (30 S at 95 °C, 30 S at 62 °C, and 30 S at 72 °C). Sanger sequencing was done using the BigDye Terminator v3.1 Cycle Sequencing Kit (Life Technologies; Thermo Fisher Scientific, Shanghai, China) on the ABI Sequencer 3500XL PE (Applied Biosystems, CA, USA).

## Results

### Pompe disease evaluation/screening

At age 51, the α-1, 4-glucosidase activity of the proband (II-5) was 6.7 in units of µmol/L/h, above the cut-off value (> 2.0). Therefore, Pompe disease was unlikely in the proband (II-5).

### MRI and CT scan findings

The MRI findings of the proband (II-5), performed at age 51, showed dilated cardiomyopathy. The proband (II-5) also underwent a CT scan of his lungs at age 51. Minimal ground-glass opacities were evident in the lower lobes bilaterally, with prominence on the right side. There was no evidence of other pathologic findings in the parenchyma of both lungs. Mild pleural effusion was seen bilaterally, with prominence on the right side. MRI on both thighs, performed at age 51, showed diffuse atrophy and fat deposition in the semitendinosus, semimembranosus, biceps femoris, and soleus muscles.

### Electrodiagnostic findings

The results of the electrodiagnostic testing of the proband (II-5), performed at age 51, are presented in Table 1 A-D. Based on the provided nerve conduction study (NCS) and electromyography (EMG) results indicate diminished amplitudes, mildly decelerated conduction velocities, and increased insertional activity, positive sharp waves, and fibrillation potentials in numerous muscles. So, the test concluded a chronic myopathic process with ongoing active denervation with some myotonic discharges.

**Table 1A:** Sensory NCS

**Nerve / Sites**	**Rec. site**	**Onset last** **ms**	**Peak Ampl** **µV**	**Peak lat** **ms**	**Distance** **cm**	**Velocity** **m/s**
L. Median – Digit II						
1. Wrist	II	2.45	27.6	3.50	15	61.2
L Ulnar – Digit V						
1. Wrist	V	2.40	35.1	3.10	12	50.0
L Sural – Lat Malleolus						
1.		2.60	7.0	3.55	12	46.2
R SURAL – Lat Malleolus						
1.		3.55	8.3	4.15	15	42.3

**Table 1B:** Motor NCS

**Nerve / sites**		**Latency** **ms**	**Ampl** **mV**	**Distance** **cm**	**Velocity** **m/s**	
L Median – APB						
1. Wrist		3.80	5.6			
2. Elbow		7.55	5.4	2.3	61.3	
L. Ulnar – ADM						
1. Wrist		2.95	9.3			
2. B. Elbow		6.90	8.9	20		50.6
3. A. Elbow		8.95	9.0	10		48.8
L Tibial (Knee) – AH						
1. Ankle		4.35	10.8			
2.Knee		13.85	8.8	38		40.0
R Tibial (Knee) – AH						
1. Ankle	4.05		9.9			
2 Knee	14.05		7.2	40		40.0
L Comm peroneal – Tib Ant						
1. Fib Head		3.35	4.8			
2. Knee		5.70	3.7	12		51.1

**Table 1C:** F Wave

**Nerve**	**Min F Lat** **ms**	**Max Lat** **ms**	**Mean Flat** **ms**
L TIBIAL (KNEE) - AH	52.15	57.40	54.73

**Table 1D:** Needle EMG

EMG Summary Table	Spontaneous					MUAP			Recruitment
	IA	Fib	PSW	Fase	Myotonic disch	Amp	Dur	PPP	Pattern
L. Thor pspinals	N	2+	2+	None	1+	1-	1-	1+	1+
L. First d inteross	N	1+	1+	None	1+	1-	1-	1+	N
L. Flex carpi rad	N	1+	1+	None	1+	1-	1-	1+	1+
L. Biceps	N	2+	1+	None	1+	2-	3+	1+	1+
L. Deltoid	N	1+	1+	None	1+	1-	1-	1+	1+
L. Iliopsoas	N	2+	2+	None	1+	2-	2-	1+	1+
L. Vast lateralis	N	1+	2+	None	1+	1-	1-	1+	1+
L. Tib anterior	N	1+	1+	None	1+	1-	1-	1+	No Activity
L. Gastrocn (MED)	N	1+	1+	None	1+	1-	1-	1+	1+

NCS, nerve conduction study; AMP, amplitude; MUAP, motor unit action potential; IA, insertion activity; Fib, fibrillation; PSW, positive sharp waves; Dur, durations; PPP, polyphasic potential; L, left; R, right

### Pathology

**Fig. 2:**
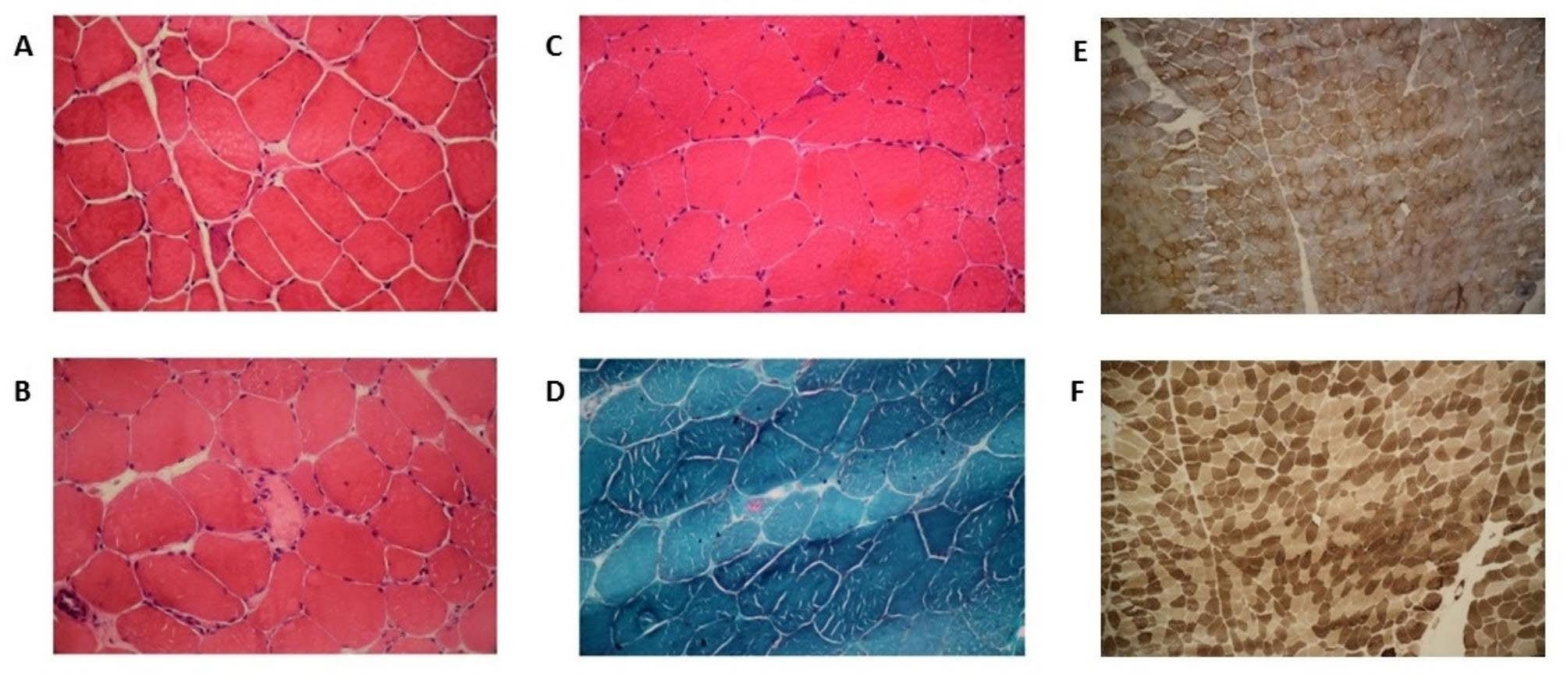
The image shows a muscle biopsy from the left vastus lateralis. **(A)** Fiber size variation with round and angular atrophic fibers and increased internalization of nuclei (H&E X200). **(B)** One necrotic fiber with myophagocytosis (H&E X200). **(C)** Subsarcolemmal basophilic aggregate in only one fiber (H&E X200). **(D)** Red-rimmed vacuole (Modified Gomori Trichrome X200). **(E)** Checkerboard pattern with no COX-negative fiber (COX + SDH x100). **(F)** Type 2 fibers predominance and slight atrophy with no fiber type grouping (ATPase 9.4 × 100)

Cytological evaluation of the pleural effusion of the proband (II-5) revealed some reactive and bland-looking isolated and clustered mesothelial cells admixed with some lymphocytes and red blood cells in a proteinaceous background. No malignant cells were detected. A muscle biopsy taken from the left vastus lateralis at age 51 years demonstrated mild myopathic atrophy. Hematoxylin and eosin staining showed striated muscle tissue with variation in fiber size. Atrophic fibers were round or angular and dispersed (Fig. 2A). Rare dispersed necrotic fibers were seen (Fig. 2B). Only one fiber showed a subsarcolemmal aggregate of homogenous basophilic materials. Internalized nuclei were increased (Fig. 2C). There was neither fibrosis nor inflammation. Modified Gomori trichrome stain revealed a few ragged red fibers and rare red-rimmed cytoplasmic vacuoles (Fig. 2D). NADH-TR reaction demonstrated good differentiation of muscle fibers with slight nonspecific intermyofibrillar network abnormalities as some uneven cytoplasmic staining.SDH reaction illustrated a few fibers with abnormal mitochondrial proliferation. COX + SDH reaction revealed no COX-negative fiber (Fig. 2E). ATPase reactions PH 9.4, 4.63, and 4.35 revealed type 2 fiber predominance, and no fiber type grouping was seen. Atrophic fibers were mostly type 1, but no fiber type disproportion was detected (Fig. 2F).

### Genetic investigations

After the filtration of the WES data, a novel heterozygous missense mutation in exon 16 of the *MYH7* (NM_000257): c.C1888A: p.Pro630Thr, was identified, which was probably responsible for MSM with dilated cardiomyopathy in this family. Sanger sequencing confirmed the presence of the c.C1888A variant in the affected proband (II-5). Both his unaffected daughter (Fig. 1A: III-1) and unaffected son (Fig. 1A: III-2) were heterozygous for this locus (Fig. 1B). Position 630 in the MYH7 protein is highly conserved among multiple species (Fig. 1E), and the missense mutation results in an amino-acid substitution from Proline to Threonine at this position.

A schematic illustration of the myosin protein and its domains is presented in Fig. 1C. The p.Pro630Thr mutation occurred within the head domain. The 3D structures of the protein representing the wild type in contrast with the mutated p.Pro630Thr are depicted in Fig. 1D. According to the American College of Medical Genetics and Genomics 2015 (ACMG) [28], c.C1888A is determined as a likely pathogenic variant (i.e., Criteria: PVS1, PM1, PM2, and PP2). The missense mutation was supported as the cause of the disease by CADD, SIFT, PolyPhen-2, PROVEAN, FATHMM, and GERP^++^.

## Discussion

Mutations in *MYH7* encoding for the β-MyHC are a common cause of hypertrophic or dilated cardiomyopathy, Laing distal myopathy, and MSM. *MYH7* maps in tandem on human chromosome 14 with *MYH6*. The *MYH7* gene is composed of 40 exons. In particular, mutations that cause MSM are located in exons 37–40 of *MYH7* [29, 30].

In this study, we analyzed three generations of an Iranian family with suspected myopathy using WES to detect the causative mutation. We found that the proband (II-5) in the family carried a heterozygous c.C1888A: p.Pro630Thr variant in the *MYH7* gene associated with MSM. The proband (II-5) had two children. The two unaffected siblings, III-1 (the proband’s daughter) and III-2 (the proband’s son), carried the same c.C1888A: p.Pro630Thr variant in the *MYH7* gene. However, no symptoms of the disease have been witnessed in them thus far, highlighting the importance of discussing disease penetrance during genetic counseling. There was no family history of muscle weakness in the remaining family members. The findings suggested that the c.C1888A: p.Pro630Thr variant could be a *de novo* variant that appeared to have occurred in the affected proband (II-5). Nonetheless, because DNA was not available from his deceased parents, we could not prove it. The recurrent independent emergence of *MYH7* mutations in different ethnic backgrounds is thought to be associated with the high prevalence of *de novo* mutations in the *MYH7* gene [31, 32].

Age at the onset of initial symptoms is usually infancy or childhood with variable penetrance. Still, it has been reported that in some patients, symptoms emerge in adult life or some may even be asymptomatic in their 40s. Li et al. reported two cases including a 46-year-old man with late-onset proximal weakness and his 26-year-old son showing talipes cavus and calf pseudohypertrophy [33]. Bohlega et al. reported three generations of a Saudi Arabian family, with the index patient who experienced the first symptoms around age 40 and her offspring in early childhood [17]. These reports indicate the intrafamilial heterogeneity on both clinical manifestations and age at onset. In the present study, the proband (II-5), a 51-year-old man, manifested his initial symptoms at around 47 years of age with no other affected relatives. Although cardiomyopathy is typically not present in MSM, we observed dilated cardiomyopathy in the proband (II-5).

Predominantly, mutations existing within the globular head of MyHC I have been associated with hypertrophic and dilated cardiomyopathies [34], whereas mutations located in the distal rod region of the protein, including Leu1793Pro, Arg1845Trp, Glu1886Lys, and His1901Leu, have been reported to cause MSM [3, 35]. Nevertheless, this final distinction is not very pertinent since there have been several reports of cases with skeletal myopathies and mutations in the globular head region [36], often representing associated cardiac involvement [9, 37]. On the other hand, numerous reports have described cardiomyopathy and mutations in the COOH-tail region of the protein [3, 14, 38, 39]. Of note, the c.C1888A: p.Pro630Thr variant, which we detected in our study and deemed culpable for MSM, is located in the myosin globular head domain.

Remarkably, different phenotypes have been found to be associated with various mutations of the same amino-acid residue of β-MyHC [32]. The missense mutation, p.Leu1793Pro, is known to cause MSM [12], whereas the heterozygous deletion at this position (pLeu1793del) was reported in a boy with distal myopathy who had undergone heart transplantation at age 3 [40]. Contrarily, the same mutation at the residue can lead to either MSM or Laing early-onset distal myopathy [23, 41]. While it remains largely unexplained why myopathy associated with *MYH7* mutations presents a variable phenotypic expression, Tasca et al. suggested that it could be due to the effect of genetic or environmental modifiers [41]. For instance, skeletal muscle fiber type proportions in humans are different based on race [42] and are influenced by both environmental and inherited factors [43]. Differences in disease severity and phenotypes can also result in variation in the ratio of mutant-to-wild type protein [44]. Proteins that interact with myosin tail like titin, alpha actinin, myomesin, M-protein, and desmin show candidate genes to modulate MSM clinical phenotypes and genetic penetrance [9].

A drosophila MSM model has recently been described to study the effects of Leu1793Pro, Arg1845Trp, and Glu1883Lys MSM mutant myosins expressed in an indirect flight and jump muscle myosin null background. Mutant animals showed highly compromised jump and flight ability. The indirect flight structure displayed myofibrillar disarray and degeneration with hyaline-like inclusions. It was demonstrated that the mutant myosin had both decreased ability to polymerize and reduced stability [45, 46]. Dahl-Halvarsson et al. expressed mutated myosin proteins in cultured human muscle cells to evaluate the impact of four missense mutations—namely Leu1793Pro, Arg1845Trp, Glu1883Lys, and His1901Leu—on myosin assembly and muscle function and assess the mechanisms leading to protein aggregation in MSM. The results indicated that the Arg1845Trp and His1901Leu mutants were prone to the formation of myosin aggregates without assembly into striated sarcomeric thick filaments [47]. On the whole, available data suggest that changes in the structural, rather than functional, properties of MyHC I caused by a mutation in the *MYH7* gene may exhibit the primary trigger of MSM [47]. Further research is needed to explain the pathogenic basis of MSM, which may play a crucial role in clinical decision-making as well as diagnostic and therapeutic development.

Clinical and genetic characteristics of at-risk individuals and carrier screening can provide more information about genetic counseling for future pregnancies in this family which will improve their quality of life. Moreover, a population study for the frequency of the p.Pro630Thr variant is currently still required. The limitations of our study are the lack of clinical and genetic data of individuals at risk of having inherited the *MYH7* variant within the family and also the confirmed carriers.

## Conclusions

An accurate diagnosis of myopathy requires information from muscle MRI and/or muscle biopsies, in tandem with complete examinations of clinical phenotypes as well as respiratory and cardiac evaluations. In the last decade, the NGS molecular technology has provided a greater discovery power to detect novel or rare variants even if clinical information is limited. In the present study, WES enabled us to make a possibly diagnosis of myopathy as MSM caused by a mutation in the *MYH7* gene.

### Electronic supplementary material

Below is the link to the electronic supplementary material.

Supplementary Material 1

Supplementary Material 2

## Data Availability

The datasets generated and/or analyzed during the current study are available in the ClinVar repository[https://www.ncbi.nlm.nih.gov/clinvar/variation/VCV001023003.3/?redir=vcv]. The submission ID of the variant in ClinVar is as follows: NM_000257.4 (MYH7): c.1888 C > A (p.Pro630Thr): VCV001023003.3.
