# Supplementary material for: A novel heterozygous missense MYH7 mutation potentially causes an autosomal dominant form of myosin storage myopathy with dilated cardiomyopathy
Source: BMC Cardiovasc Disord. 2023 Oct 4;23:487. doi: 10.1186/s12872-023-03538-8 (PMC10552240; doi:10.1186/s12872-023-03538-8)
Supplement: Supplementary file 2 — Supplementary Material 2 [file 12872_2023_3538_MOESM2_ESM.docx]

**Supplements**

**Supplementary 1.** The WES fileting strategy.
